# Mathematical Modeling to Assess the Drivers of the Recent Emergence of Typhoid Fever in Blantyre, Malawi

**DOI:** 10.1093/cid/civ710

**Published:** 2015-10-07

**Authors:** Virginia E. Pitzer, Nicholas A. Feasey, Chisomo Msefula, Jane Mallewa, Neil Kennedy, Queen Dube, Brigitte Denis, Melita A. Gordon, Robert S. Heyderman

**Affiliations:** 1 Departmentof Epidemiology of Microbial Diseases, Yale School of Public Health, New Haven, Connecticut; 2 Malawi-Liverpool-Wellcome Trust Clinical Research Programme, University of Malawi College of Medicine, Blantyre; 3 Liverpool School of Tropical Medicine, United Kingdom; 4 Department of Microbiology; 5 Department of Medicine; 6 Department of Paediatrics and Child Health, University of Malawi College of Medicine, Blantyre; 7 Institute of Infection and Global Health, University of Liverpool; 8 Division of Infection and Immunity, University College London, United Kingdom

**Keywords:** transmission dynamics, *Salmonella* Typhi, H58 haplotype, multidrug resistance

## Abstract

***Background.*** Multiyear epidemics of *Salmonella enterica* serovar Typhi have been reported from countries across eastern and southern Africa in recent years. In Blantyre, Malawi, a dramatic increase in typhoid fever cases has recently occurred, and may be linked to the emergence of the H58 haplotype. Strains belonging to the H58 haplotype often exhibit multidrug resistance and may have a fitness advantage relative to other *Salmonella* Typhi strains.

***Methods.*** To explore hypotheses for the increased number of typhoid fever cases in Blantyre, we fit a mathematical model to culture-confirmed cases of *Salmonella enterica* infections at Queen Elizabeth Central Hospital, Blantyre. We explored 4 hypotheses: (1) an increase in the basic reproductive number (*R*_0_) in response to increasing population density; (2) a decrease in the incidence of cross-immunizing infection with *Salmonella* Enteritidis; (3) an increase in the duration of infectiousness due to failure to respond to first-line antibiotics; and (4) an increase in the transmission rate following the emergence of the H58 haplotype.

***Results.*** Increasing population density or decreasing cross-immunity could not fully explain the observed pattern of typhoid emergence in Blantyre, whereas models allowing for an increase in the duration of infectiousness and/or the transmission rate of typhoid following the emergence of the H58 haplotype provided a good fit to the data.

***Conclusions.*** Our results suggest that an increase in the transmissibility of typhoid due to the emergence of drug resistance associated with the H58 haplotype may help to explain recent outbreaks of typhoid in Malawi and similar settings in Africa.

Typhoid fever, caused by infection with the human-restricted bacterial pathogen *Salmonella enterica* serovar Typhi, is a major cause of illness and mortality in regions of the world with limited access to improved water and sanitation [1]. Recent estimates have placed the global burden of typhoid fever at 11.9–26.9 million cases and 129 000–270 000 deaths per year [2–5]. Although the endemic burden of typhoid fever in South and Southeast Asia has long been recognized, less is known about the burden of disease in sub-Saharan Africa.

In Malawi, as in much of sub-Saharan Africa, nontyphoidal *Salmonella* (NTS) serovars have been a much more common cause of bloodstream infections over the past 2 decades. Sequential epidemics of *Salmonella enterica* serovars Typhimurium and Enteritidis have been observed [6]. *Salmonella* Typhi represented just 2% (105/5061) of *Salmonella* isolates detected by sentinel surveillance at Queen Elizabeth Central Hospital (QECH) in Blantyre, the largest hospital in Malawi, between 1998 and 2004 [6]. Since 2011, however, there has been a substantial increase in the number of confirmed cases of *Salmonella* Typhi at QECH. The number of typhoid cases increased from 14 per year during 1998–2010 to 843 cases in 2013. This epidemic of typhoid fever has continued for at least 3 years [7] and coincides with numerous reports of ongoing epidemics of typhoid fever in settings across Africa [7–12]. These epidemics have closely followed the recent global emergence of the H58 haplotype of *Salmonella* Typhi [13]. The H58 lineage is highly clonal and differentiated from other haplotypes using a simple single nucleotide polymorphism–based typing scheme [14], and is associated with high levels of multidrug resistance [13, 15].

There are a variety of hypotheses that could explain the large increase in typhoid fever cases in Blantyre, including (1) an increase in population density in Blantyre beyond a critical threshold for transmission; (2) waning heterotypic immunity to *Salmonella* Enteritidis that is cross-protective against *Salmonella* Typhi; (3) an increase in the prevalence of multidrug resistant (MDR) strains, resulting in prolonged persistence; and (4) the emergence of the MDR H58 haplotype, which has become dominant in many places around the world and may be more transmissible than other strains [13]. The latter 2 hypotheses are tightly coupled, but describe slightly different mechanisms by which the H58 haplotype, and MDR strains more broadly, may have led to the outbreak.

Mathematical models provide a platform for assessing the plausibility of these hypotheses to help direct future research efforts. We therefore adapted a mathematical model for the transmission dynamics of typhoid developed previously [16] by fitting to age-specific data on microbiologically confirmed cases of typhoid fever in Blantyre, Malawi. We modified the model to explore the 4 hypotheses outlined above, and examined whether each of them could explain the recent prolonged outbreak of typhoid fever cases in this setting.

## METHODS

### Data Sources

We utilized data on microbiologically confirmed cases of *Salmonella* infection among patients seeking care at QECH in Blantyre, Malawi. QECH is the largest government hospital in Malawi, providing free care for patients from Blantyre district and the surrounding region. The Malawi-Liverpool-Wellcome Trust Clinical Research Programme (MLW) has conducted routine surveillance for bloodstream infections among all adults (≥16 years) admitted with fever >37.5°C and febrile children (<16 years) who are malaria slide negative or critically ill, and all afebrile patients with clinical suspicion of sepsis since 1996, with ad hoc surveillance prior to 1998. The data and sample processing has been described previously [6, 7, 17]. We had data on the aggregate number of *S. enterica* isolates by serovar from January 1996 to February 2015, as well as data on *Salmonella* Typhi isolates by patient age (in 5-year age groups) since February 2010.

We also obtained demographic data on the population of Blantyre district (Blantyre city + Blantyre rural) on a monthly basis between June 1999 and June 2011 from the Malawi National Statistical Office [18]. Data on the crude annual birth and death rate in Malawi from 1950 to 2030 (estimated) were obtained from the United Nations World Population Prospects database [19], and the age distribution of the population in the Southern region (in 5-year age groups) was obtained from the 2008 Malawi census [18].

### Model Description

We adapted a previously developed mathematical model for the transmission dynamics of typhoid in South Asia [16] by fitting to the data from QECH and modifying the model to explore hypotheses for the increase in typhoid fever cases beginning in 2010.

**Figure 1.**
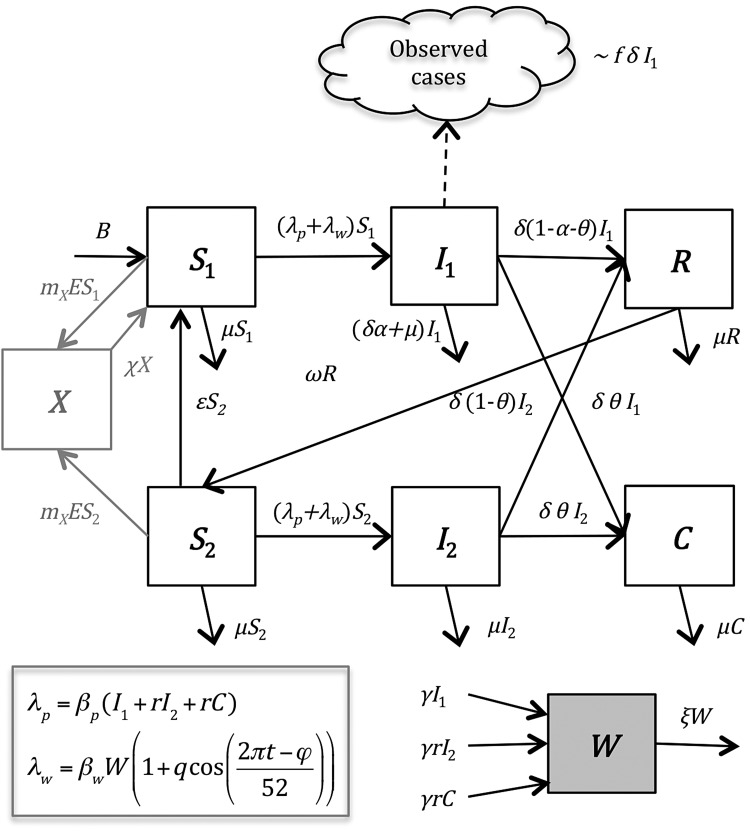
Compartmental diagram of model structure. The model is described in the “Methods” section (“Model Description”).

The model is illustrated in Figure 1. In brief, the model assumes that individuals are born susceptible to infection and disease caused by *Salmonella* Typhi (*S*_1_). Susceptible individuals are infected at a rate (*λ_p_* + *λ_w_*) that is mediated through contaminated food and water in the immediate environment (“short-cycle transmission,” *λ_p_*) as well as bacterial contamination of water supplies and the broader environment (“long-cycle transmission,” *λ_w_*). Individuals experiencing primary infection (*I*_1_) remain infectious for a period 1/*δ*, after which we assume a fraction (*α*) experience disease-induced mortality, a fraction (*θ*) develop an infection of the gall bladder and become chronic carriers (*C*), and the remaining individuals (1-*θ*-*α*) recover and are temporarily immune (*R*). We assume that *θ* depends on the age of the infected individual, consistent with epidemiological data [20]. Immunity to reinfection wanes at a rate *ω*, leaving the individual partially susceptible (*S*_2_). If reinfection occurs prior to waning of partial immunity (at a rate *ϵ*), we assume these reinfections (*I*_2_) are subclinical and are not reflected in our data. Subclinically infected individuals can again recover or become chronic carriers. We assume all infectious individuals shed bacteria into the environmental reservoir (*W*) at a rate *γ*, and the bacteria remain infectious for a period 1/*ξ*. Both chronic carriers and subclinically infected individuals have infectiousness reduced by a factor *r*. For all models, we assumed that short-cycle transmission was frequency dependent and mixing was homogeneous; we explored both frequency- and density-dependent long-cycle transmission depending on the model scenario.

In preliminary analyses, we noted that the seasonality of typhoid isolates at QECH was significantly correlated with seasonal variability in rainfall at lags of 6–21 weeks (*P* < .05). We postulated that this correlation reflects an association between rainfall and the rate of long-cycle transmission. Therefore, we assumed that the environmental transmission parameter *β_w_* varied seasonally with rainfall, with peak transmission occurring at the time of peak rainfall (see Supplementary Methods and Supplementary Figure 1).

The fixed model parameters and their sources are described in Table 1, and the estimated parameters are described in Table 2.

**Table 1.** Fixed Model Parameters

Parameter Definition	Symbol	Value	Source
Birth rate	*B*	31.3–55.0 live births per 1000 per year	Census data
Natural mortality rate	*μ*	7.7–27.8 deaths per 1000 per year	Census data
Duration of infectiousness	1/*δ*	4 wk	[23]
Seasonal offset parameter (timing of seasonal peak)	*ϕ*	4.9 wk	Based on peak in seasonal rainfall^a^
Fraction infected who become chronic carriers	*θ*	0.003–0.101 depending on age	[20]
Disease-induced mortality	*α*	0.001	Assumption [5]
Duration of temporary full immunity to infection	1/*ω*	104 wk	Assumption^b^ [24]
Rate of shedding into the water supply	*γ*	1 infectious unit/wk	Assumption^c^
Rate of decay of infectious particles from water supply	*ξ*	1/3 wk^−1^	[25]

^a^ See Supplementary Methods.

^b^ Model fit is not sensitive to this parameter [16].

^c^ Nonidentifiable/inseparable from the estimated long-cycle transmission parameter, *β_w_*.

**Table 2.** Model Parameter Estimates and Bayesian Information Criteria for Best-Fit Models for Each Scenario

Parameter Definition	Symbol	Prior Distribution	Scenario 1	Scenario 2	Scenario 3	Scenario 4
Basic reproductive number for short-cycle transmission	*R_0,p_*	Uniform (0,10)	0.61	1.6	0.04	1.6
Basic reproductive number for long-cycle transmission	*R_0,w_*	Uniform (0,10)	0.71, 2.2^a^	4.7	5.5	2.1
Amplitude of seasonal forcing (long-cycle transmission)	*q*	Uniform (0,1)	0.41	0.37	0.26	0.53
Rate of waning immunity to clinical disease (years^−1^)	*ϵ*	Uniform (0,2)	3.4 × 10^−6^	1.5	3.6 × 10^−7^	1.0 × 10^−6^
Relative infectiousness of chronic and short-term carriers	*r*	Uniform (0,1)	0.047	0.068	0.43	0.26
Reporting fraction	*f*	Uniform (0,1)	0.0087	0.0053	0.0029	0.0027
Proportionality factor between incidence of *Salmonella* Enteritidis infection and observed incidence of invasive disease	*m_X_*	Uniform (1,*N*^b^)	…	1500	…	…
Duration of cross-immunity, wk	1/*χ*	Uniform (1, 1000)	…	993	…	…
Beginning week of increase in the duration of infectiousness or transmission rate	*t*_0_	Uniform (0,*L*^c^)	…	…	27 February 2011	21 November 2010
End week of increase in duration of infectiousness or transmission rate	*t*_1_	Uniform (0,*L*^c^)	…	…	13 March 2013	10 March 2013
Magnitude of increase in duration of infectiousness or transmission rate	*m*	Uniform (1,10)	…	…	2.3	3.0
Bayesian information criteria			6626	6242	5941	5985

^a^ *R*_0,*w*_ varied with population size in scenario 1; the values listed correspond to the range of *R*_0,*w*_ between January 1996 and February 2015.

^b^ *N* represents the total population size in 2000 (870 000).

^c^ *L* represents the length of the time series (993 weeks); estimated value was rounded to the nearest week and the corresponding calendar date of that week is given.

### Basic Reproductive Number (*R*_0_) and the Role of Chronic Carriers

The basic reproductive number of the model is given by the equation

$$R_{0}=\frac{1}{\delta +\mu }\left(\beta_{p}+\frac{\gamma \beta_{w}}{\xi }\right)\left(1+\frac{\delta \theta r}{\mu }\right),$$

which is defined as the expected number of secondary infections produced by an infectious individual in a fully susceptible population [16]. It is possible to rewrite *R*_0_ as the sum of the basic reproductive numbers of short-cycle (*R*_0,*p*_) and long-cycle (*R*_0,*w*_), where

$$R_{0,p}=\frac{\beta_{p}}{\mu +\delta }\left(1+\frac{\delta \theta r}{\mu }\right)$$

and

$$R_{0,w}=\frac{\gamma \beta_{w}}{\xi (\mu +\delta )}\left(1+\frac{\delta \theta r}{\mu }\right).$$

The proportion of transmission from chronic carriers vs acute primary infections (*c_p_*) can be calculated as

$$c_{p}=\frac{\delta \theta r}{\mu +\delta \theta r}.$$

### Model Scenarios

To examine whether increasing population density in Blantyre district alone can explain the pattern of typhoid emergence at QECH (scenario 1), we assumed that long-cycle transmission was density dependent, such that the rate of transmission would increase as the population increased. We estimated the rate of transmission from short-cycle and long-cycle transmission, amplitude of seasonality in long-cycle transmission, rate of waning partial immunity, relative infectiousness of carriers, and a “reporting fraction” necessary to scale the predicted number of clinical infections in Blantyre district to the observed weekly number of culture-confirmed cases at QECH (Table 2).

To explore the hypothesis that the immune interaction with other *Salmonella* serovars, particularly *Salmonella* Enteritidis, could explain the observed pattern (scenario 2), we assumed that susceptible individuals could develop cross-protective immunity (*X*) at a rate proportional to the observed number of *Salmonella* Enteritidis isolates (Figure 1). Individuals who lost this cross-protective immunity became fully susceptible to clinical infection (conservative assumption). We estimated both the proportionality factor (*m_X_*) and the duration of cross-immunity (1/*χ*), in addition to the other 6 parameters described above (Table 2).

We modeled the impact of an increase in MDR typhoid by allowing for an increase in the duration of infectiousness (scenario 3). We assumed that the duration of infectiousness was 3 weeks (on average) with access to effective antibiotic therapy, but increased linearly following the emergence of MDR strains; we estimated the week this increase began (*t*_0_), the week it ended (*t*_1_ ≥ *t*_0_), and the magnitude of the increase (*m*), in addition to the 6 parameters described in scenario 1 (Table 2).

Finally, we modeled an increase in the fitness of *Salmonella* Typhi associated with the emergence of the H58 haplotype, possibly due to a higher in vitro growth rate [21]. We allowed for an increase in the rate of both short- and long-cycle transmission (scenario 4). Again, we estimated both the week this increase began (*t*_0_), the week it ended (*t*_1_ ≥ *t*_0_), and the magnitude of the increase (*m*), assuming a linear increase in both *β_p_* and *β_w_* between *t*_0_ and *t*_1_, in addition to the other 6 parameters (Table 2).

For scenarios 2–4, we assumed frequency-dependent long-cycle transmission, and examined the sensitivity of our results to this assumption (Supplementary Table 1).

### Model Fitting

We fit our model to the data on culture-confirmed cases of *S.* Typhi at QECH from 1996 to 2015 by maximum a posteriori estimation. We assumed uniform prior distributions for all model parameters (Table 2). We calculated the log-likelihood of each model assuming that the weekly number of observed cases in each age group is Poisson-distributed with a mean equal to the model-predicted number of clinical infections over the duration of infectiousness times the reporting fraction, *f*. Because we only had data on the age of cases beginning in 2010, we fit the model to the data up to February 2010 by summing the model-predicted number of cases each week across all age groups. More details on the model-fitting procedure can be found in the Supplementary Methods. We calculated the Bayesian information criterion (BIC) for each best-fit model to compare models with a different number of estimated parameters across the different scenarios (Table 2).

## RESULTS

**Figure 2.**
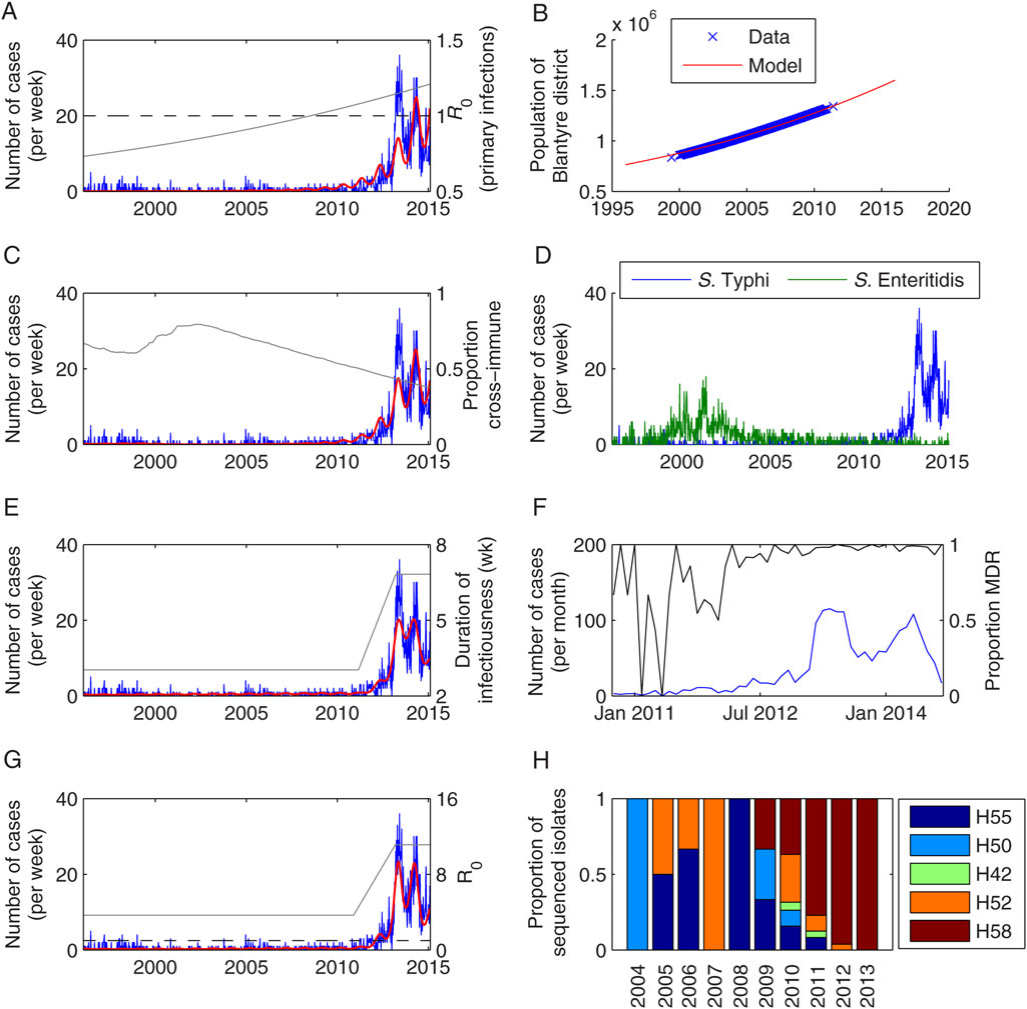
Fit of models for scenarios 1–4 to data on weekly number of culture-confirmed *Salmonella* Typhi infections at Queen Elizabeth Central Hospital in Blantyre, Malawi, from January 1996 to February 2015. *A*, Observed weekly number of typhoid fever cases (blue) and best-fit model for scenario 1 (red). The *R*_0_ for primary infections is plotted in gray, while the dashed black line represents *R*_0_ = 1. *B*, Observed (blue) and modeled (red) population size of Blantyre district (in millions). *C*, Observed weekly number of typhoid fever cases (blue) and best-fit model for scenario 2 (red). The proportion of the population with cross-immunity from past *Salmonella* Enteritidis infection is plotted in gray. *D*, Observed weekly number of *Salmonella* Typhi (blue) and *Salmonella* Enteritidis (green) cases. *E*, Observed weekly number of typhoid fever cases (blue) and best-fit model for scenario 3 (red). The duration of infectiousness (in weeks) is plotted in gray. *F*, Observed monthly number of *Salmonella* Typhi cases (blue) and the proportion of isolates exhibiting multidrug resistance (MDR) (black) from October 2010 to December 2014. *G*, Observed weekly number of typhoid fever cases (blue) and best-fit model for scenario 4 (red). The overall *R*_0_ is plotted in gray. *H*, The proportion of sequenced *Salmonella* Typhi isolates belonging to each haplotype by year.

For scenario 1, the best-fit model predicted that the number of *Salmonella* Typhi cases per year would increase from 2.8 in 1996 to 41.3 in 2009, after which the number of cases approximately doubled each year until there were 879 cases in 2014 (Figure 2*A*). We estimated that the basic reproductive number, *R*_0_, increased from 1.3 in 1996 to 2.8 in 2015. Chronic carriers were estimated to account for 45%–57% of transmission, such that the *R*_0_ of primary infections alone increased from 0.73 to 1.2, crossing the critical threshold of *R*_0_ = 1 in July 2008 (Figure 2*A*). Approximately 54%–78% of transmission was estimated to occur via the long cycle. However, this model failed to capture the sharp rise in cases in 2013 (Figure 2*A*).

The best-fit model under scenario 2 predicted a similar increase in the number of typhoid cases per year from a range of 2.5–29.1 between 1996 and 2009 (with slightly more cases in the late 1990s) to 1076 in 2014. However, the model again failed to capture the peak in cases in 2013 (Figure 2*C*). The basic reproductive number was estimated to be *R*_0_ = 7.0 under this scenario, with 75% of transmission occurring via the long cycle. The incidence of cross-immunizing infection with *Salmonella* Enteritidis was estimated to be 1500-fold the observed number of invasive cases presenting to QECH, with immunity lasting 993 weeks on average (Table 2).

If we assumed the duration of infectiousness increased coincident with the emergence of MDR *Salmonella* Typhi (scenario 3), the model did a better job of capturing the observed pattern of typhoid cases at QECH. The model predicted 17.0–33.7 cases per year prior to 2010, when incidence began to increase sharply, with a peak of 21.5 cases per week at the height of the epidemic in 2013 (Figure 2*E*). The average duration of infectiousness was estimated to increase from 3 weeks prior to February 2011 to 6.8 weeks after March 2013, which is similar to the timing of the observed increase in the prevalence of MDR *Salmonella* Typhi isolates (Figure 2*F*) [7].

When we allowed for an increase in the transmission rate associated with the emergence of the H58 haplotype (scenario 4), the model was again able to reproduce the observed pattern of typhoid cases both before and after 2010, although it provided a slightly worse fit compared to scenario 3 according to BIC (Table 2). The predicted number of cases increased from 13.8–24.6 cases per year during 1996–2009 to 837 in 2013 and 708 in 2014 (Figure 2*G*). The basic reproductive number was estimated to increase from *R*_0_ = 4.3 prior to September 2010 to 12.9 after January 2013. This is similar to the timing of the emergence of the H58 haplotype in Blantyre, which was first detected in 2009 and constituted 100% of sequenced isolates from 2013 (Figure 2*H*) [7]. The *R*_0_ for primary infections was <1 prior to March 2012; thus, chronic carriers were estimated to drive much of the transmission (*c_p_* = 90%) to explain the prolonged period of low reported cases in the late 1990s/early 2000s (Supplementary Figure 3).

**Figure 3.**
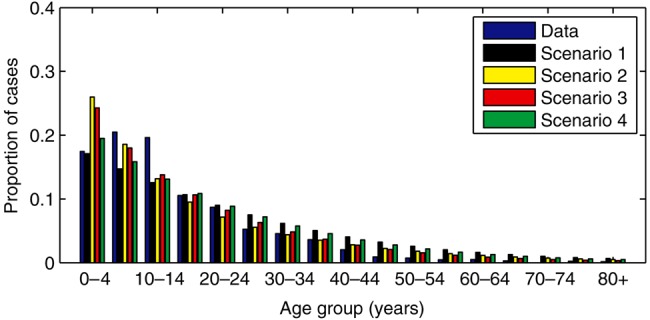
Age distribution of culture-confirmed *Salmonella* Typhi infections at Queen Elizabeth Central Hospital in Blantyre, Malawi. The observed proportion of cases in each age group diagnosed between October 2010 and February 2015 is indicated by the blue bars, while the other bars represent the model-predicted age distribution of cases for the corresponding time period for the best-fit models under scenario 1 (black), scenario 2 (yellow), scenario 3 (red), and scenario 4 (green).

All of the models provided a reasonably good fit to the age distribution of cases between 2010 and 2015, but tended to underestimate the proportion of cases in those aged 5–15 years and slightly overestimate the proportion of cases in those aged 0–5 years and/or the older (≥25 years) age groups (Figure 3).

**Figure 4.**
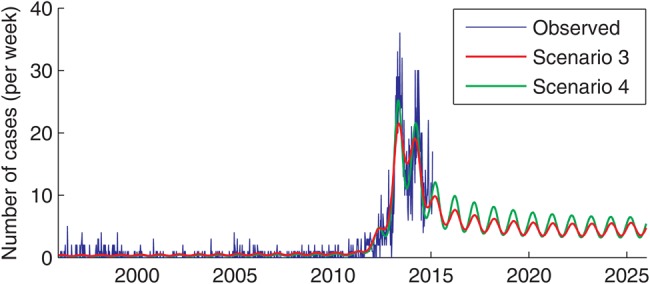
Model projection of weekly number of culture-confirmed *Salmonella* Typhi infections at Queen Elizabeth Central Hospital in Blantyre, Malawi, 1996–2025.

If we use the best-fit model from scenario 3 to project the future number of typhoid cases presenting to QECH (assuming status quo), the model predicts that typhoid incidence will decline over the next 10 years relative to the peak in 2013–2014 (Figure 4). However, there will still be an estimated 230–399 cases per year, which is considerably greater than observed prior to 2010. Predictions are similar for the best-fit model under scenario 4 (Figure 4).

## DISCUSSION

Epidemics of typhoid fever lasting several years have recently been reported in countries across eastern and southern Africa, including Zimbabwe [8], Zambia [10], Kenya [9], Uganda [11], Mozambique [12], and, most recently, Malawi [7]. This stands in contrast to the long-established endemic burden of typhoid in South and Southeast Asia [1, 5, 22]. In Blantyre, Malawi, longitudinal surveillance demonstrate that *S*. Typhi was an uncommon cause of bloodstream infections prior to 2011, and that an increase of MDR typhoid was initially due to a wide range of clades subsequently dominated by the H58 lineage [13]. A variety of hypotheses have been proposed to explain the emerging epidemics of typhoid fever in Africa. Our analysis supports the idea that these epidemics, particularly the epidemic in Blantyre, was primarily caused by the emergence of MDR and the recent introduction of the H58 haplotype to Africa.

Mathematical modeling provides a tool for testing hypotheses to explain patterns in data. Our best-fit models are able to reproduce the sharp rise in typhoid fever cases at QECH between 2011 and 2013. The models suggest that prior to 2010, typhoid transmission in Blantyre was driven by a combination of transmission from chronic carriers (whose prevalence was predicted to be 0.3%–1%) and subcritical (*R*_0_ < 1) transmission from primary infections. As the duration of infectiousness (and/or transmission rate) of typhoid increased following the emergence of MDR strains and the H58 haplotype in particular, primary infections were capable of infecting >1 other person on average, leading to exponential epidemic growth. However, our models predict that the typhoid epidemic in Blantyre will begin to subside over the next couple years as fewer susceptible people remain in the population, although case counts will remain higher than in the 1990s–2000s.

Interestingly, the best-fit models under each of the scenarios estimated that chronic carriers played a very different role in transmission. The estimated proportion of transmission due to chronic carriers varied from 45% under scenario 1 to 95% under scenario 3, although acute infections drove the increased force of infection (ie, rate of infection per susceptible individual) during the epidemic period (Supplementary Figure 3). Previous models have identified this as a key source of uncertainty in mathematical models for typhoid, which could have a substantial impact on the level of indirect protection expected from vaccination [16]. Studies are needed to determine how the prevalence of chronic carriers and the role they play in transmission varies across settings and over time.

There are a number of limitations to our analysis. Ours is a simplified model of typhoid transmission dynamics. We assume homogeneous mixing, such that everyone in Blantyre district is equally likely to acquire typhoid and transmit to anyone else. We do not account for spatial clustering of transmission, which may additionally prolong the current outbreak, or age-related differences in the risk of infection and/or diagnosis of typhoid. Hence, our model tended to underestimate the proportion of cases in 5- to 15-year-olds. We also assume that there are no specific host risk factors for typhoid, in contrast to invasive NTS, where human immunodeficiency virus, malaria, and malnutrition are prominent. Our model does not take into account the possibility that multiple exposures to *Salmonella* Typhi or cross-reactive species may be required to generate protective immunity. Although this may impact parameter estimation and prolong the time taken to reach a steady state, it is unlikely to alter our conclusions. Finally, some of the model parameters are not well identified in our fitted models. Although this does not affect our overall conclusions, it is imperative that we better understand the nature of typhoid transmission and immunity to use mathematical models to assess the potential impact of future interventions. Epidemiological and clinical studies are needed to inform our modeling efforts.

Our analysis suggests that the emergence of drug resistance and the H58 haplotype, rather than urbanization and overcrowding or changing immunological susceptibility in the population, are the most important factors explaining the current outbreak of typhoid fever in Blantyre. Whether these factors can also explain recent outbreaks in other parts of eastern and southern Africa is unclear, although the timing of the outbreaks is remarkably consistent with the phylogeography of the MDR H58 haplotype [13]. Understanding the factors that underlie the observed patterns of typhoid fever in Africa can help to guide future vaccine and nonvaccine efforts to control the disease. Mathematical models such as the one described here can be used to assess the potential impact of different vaccination strategies, accounting for both the direct and indirect protection conferred by vaccines.

## Supplementary Data

Supplementary materials are available at *Clinical Infectious Diseases* online (http://cid.oxfordjournals.org). Supplementary materials consist of data provided by the author that are published to benefit the reader. The posted materials are not copyedited. The contents of all supplementary data are the sole responsibility of the authors. Questions or messages regarding errors should be addressed to the author.

Supplementary Data
