# Supplementary material for: Mathematical Modeling to Assess the Drivers of the Recent Emergence of Typhoid Fever in Blantyre, Malawi
Source: Clin Infect Dis. 2015 Oct 7;61(Suppl 4):S251–8. doi: 10.1093/cid/civ710 (PMC4596932; doi:10.1093/cid/civ710)
Supplement: Supplementary Data [file supp_61_suppl-4_S251__index.html]

Supplementary Data 

# Mathematical Modeling to Assess the Drivers of the Recent Emergence of Typhoid Fever in Blantyre, Malawi

## Supplementary Data

Supplementary Data

- Supplementary Data - Pdf file
