# Supplementary material for: Mathematical Modeling to Assess the Drivers of the Recent Emergence of Typhoid Fever in Blantyre, Malawi
Source: Clin Infect Dis. 2015 Oct 7;61(Suppl 4):S251–8. doi: 10.1093/cid/civ710 (PMC4596932; doi:10.1093/cid/civ710)

Supplementary Material

Mathematical modeling to assess the drivers of the recent emergence of typhoid fever in Blantyre, Malawi

Virginia E. Pitzer*, *Department of Epidemiology of Microbial Diseases, Yale School of Public Health, New Haven, CT USA*

Nicholas A. Feasey, *Malawi-Liverpool-Wellcome Trust Clinical Research Programme, University of Malawi College of Medicine, Blantyre, Malawi and Liverpool School of Tropical Medicine, Liverpool, UK*

Chisomo Msefula, *Malawi-Liverpool-Wellcome Trust Clinical Research Programme, University of Malawi College of Medicine, Blantyre, Malawi and Department of Microbiology, University of Malawi College of Medicine, Blantyre, Malawi*

Jane Mallewa, *Department of Medicine, University of Malawi College of Medicine, Blantyre, Malawi*

Neil Kennedy, *Department of Paediatrics and Child Health, University of Malawi College of Medicine, Blantyre, Malawi*

Queen Dube, *Department of Paediatrics and Child Health, University of Malawi College of Medicine, Blantyre, Malawi*

Brigitte Denis, *Malawi-Liverpool-Wellcome Trust Clinical Research Programme, , University of Malawi College of Medicine, Blantyre, Malawi*

Melita A. Gordon, *Malawi-Liverpool-Wellcome Trust Clinical Research Programme, University of Malawi College of Medicine, Blantyre, Malawi and Institute of Infection and Global Health, University of Liverpool, Liverpool, UK*

Robert S. Heyderman, *Malawi-Liverpool-Wellcome Trust Clinical Research Programme, University of Malawi College of Medicine, Blantyre, Malawi and Division of Infection & Immunity, University College London, London, UK*

**Corresponding author*
E-mail: virginia.pitzer@yale.edu

Rainfall and typhoid fever cases in Blantyre, Malawi

As a preliminary analysis, we examined the association between rainfall and the occurrence of culture-confirmed typhoid fever cases at Queen Elizabeth Central Hospital (QECH) in Blantyre, Malawi. We obtained data on daily rainfall between 2001 and 2010 from the Chileka and Chichiri weather stations in Blantyre. We averaged the data from the two stations and aggregated it by week of the year. We then calculated the Pearson's correlation coefficient (ρ) between the average weekly rainfall in Blantyre and the total number of typhoid fever cases at QECH by week of the year from 1998-2014 (Figure S1a) at lags varying from 0 to 51 weeks. We found that rainfall and weekly typhoid cases were significantly and positively correlated at lags of 6 to 21 weeks (ρ>0.33, p<0.05), with the peak correlation occurring at a lag of 13 weeks (ρ=0.82).

We hypothesized that the lag between the peak in rainfall and the peak in typhoid cases could be explained by differences between when the rate of transmission from susceptible to infectious individuals ($\beta_p+\beta_w$) peaks (which is independent of the prevalence of infection) versus when the force of infection ($\lambda_p+\lambda_w$) peaks (per susceptible rate of infection, which is proportion to the number of currently infectious individuals and bacteria in the environment), as well as reporting delays associated with the time from infection to seeking care and being diagnosed with typhoid fever at QECH. Furthermore, we assumed that rainfall would primarily affect the rate of long-cycle transmission. Therefore, we assumed the peak in β_w would coincide with the peak in seasonal rainfall.

Since we did not have rainfall data for all years, and to simplify the analysis, we assumed that β_w varied according to a sinusoidal seasonal forcing function. Thus,

$$\beta_w(t) = \beta_0\left(1 + q\cos\left(\frac{2\pi t - \phi}{52.18}\right)\right)$$

where t is time in weeks, β_0 is the mean transmissibility parameter for long-cycle transmission, q is the amplitude of variation (estimated), and ϕ is the seasonal offset parameter (corresponding to the week of peak transmissibility). We estimated ϕ by fitting a cosine curve to the average weekly rainfall data (Figure S1b) using least squares, then fixed ϕ at this value for all subsequent analyses.

Model fitting procedure

We fit our model to the data on culture-confirmed cases of *S.* Typhi at QECH from 1996 to 2015 by maximum a posteriori (MAP) estimation. We assumed uniform prior distributions for all model parameters. We calculated the log-likelihood of each model assuming that the observed number of cases in week w and age group a ($C_{w,a}$) is Poisson-distributed with a mean equal to the model-predicted number of clinical infections over the duration of infectiousness times the reporting fraction ($D_{w,a} = f\delta I_{1,w,a}$):

$$LL = \sum_w \sum_a C_{w,a}\log\left(D_{w,a}\right) - D_{w,a} - \sum_{i=1}^{C_{w,a}}\log(i).$$

Since we only had data on the age of cases beginning in 2010, we fit the model to the data up to February 2010 by summing the model-predicted number of cases each week across all age groups.

We initialized each model assuming a baseline state distribution $\mathbf{P_0}$ in January 1950, then simulated the model and discarded the burn-in period prior to 1996. We assumed $\mathbf{P_0}$ = $\{\mathbf{S_1},\mathbf{I_1},\mathbf{R},\mathbf{S_2},\mathbf{I_2},\mathbf{C}\}$, where:

$$\mathbf{S_1} = \begin{cases} 0.9N_a - 10 & \text{for } a < 30 \\ 0.89N_a - 10 & \text{for } a \geq 30 \end{cases}$$

$$\mathbf{I_1} = 10 \quad \text{for all } a$$

$$\mathbf{R} = 0 \quad \text{for all } a$$

$$\mathbf{S_2} = 0.1N_a - 10 \quad \text{for all } a$$

$$\mathbf{I_2} = 10 \quad \text{for all } a$$

$$\mathbf{C_0} = \begin{cases} 0 & \text{for } a < 30 \\ 0.01N_a & \text{for } a \geq 30 \end{cases}$$

where N_a is the estimated population size in age group a in 1950. We ensured that the modeled population size and age distribution appeared consistent with the observed demographics in Blantyre district (Figure S2).

To estimate the parameters of each model, we sampled 1,000 parameter sets from a reasonable range of parameters using Latin hypercube sampling (Table 2). We calculated the log-likelihood for each sampled parameter set then minimized the negative log-likelihood of each model, minus the associated prior distributions, using a simplex search method beginning at the top ten parameter sets for each model (using the "fminsearch" command in MATLAB v7.14). The best-fit model for each scenario corresponded to the parameter set that yielded the highest posterior probability.

Finally, we calculated the Bayesian information criterion (BIC) for each best-fit model to compare models with a different number of estimated parameters across the different scenarios (Table 2).

Contribution of acute infections versus chronic carriers to the force of infection

To examine how the role of chronic carriers in transmission changes over time, we calculated the time-varying force of infection for acute infections (λ_A) versus chronic carriers (λ_C), where:

$$\lambda_A(t) = \beta_p(I_1(t) + rI_2(t)) + \beta_w(t)W\frac{I_1(t) + rI_2(t)}{(I_1(t) + rI_2(t) + C(t))N(t)},$$

$$\lambda_C(t) = \beta_p C(t) + \beta_w(t)W\frac{C(t)}{(I_1(t) + rI_2(t) + C(t))N(t)}.$$

Sensitivity to assumption of frequency- versus density-dependent transmission

In Scenario 1, we make the assumption that long-cycle transmission is density dependent in order to examine the hypothesis that increased population density in Blantyre could explain the recent epidemic of typhoid fever. As a base case, we wanted to explore the support for hypotheses underlying Scenarios 2-4 independent of Scenario 1. Therefore, we assumed long-cycle transmission was frequency dependent for Scenarios 2-4.

However, it is possible that increases in transmission related to the increasing population density may partially explain the observed pattern of typhoid cases in Blantyre in combination with one (or more) of the other hypotheses. We therefore refit the models under Scenarios 2-4 allowing for density-dependent long-cycle transmission in order to

examine the sensitivity of our conclusions to this assumption. Overall, the best-fit model parameters and overall model fits (as indicated by BIC) were similar under both frequency- and density-dependent transmission assumptions (Table 2, Table S1). Interestingly, the estimated value of r (the relative infectiousness of carriers) decreased substantially for Scenario 3, suggesting that the period of low incidence in the 1990s/2000s can be explained by either transmission from chronic carriers or lower population density.

Table S1. Model parameter estimates and Bayesian information criteria (BIC) for best-fit models for Scenarios 2-4 assuming density-dependent long-cycle transmission.

**Parameter definition**	**Symbol**	**Scenario 2**	**Scenario 3**	**Scenario 4**
Basic reproductive number for short-cycle transmission	$R_{0,p}$	2.0	2.6	3.7
Basic reproductive number for long-cycle transmission	$R_{0,w}$	0-0.55	0-0.81	0-1.1
Amplitude of seasonal forcing (long-cycle transmission)	q	0.90	1.0	0.85
Rate of waning immunity to clinical disease (years^{-1})	ε	1.5	6.2x10^{-6}	2.6x10^{-6}
Relative infectiousness of chronic and short-term carriers	r	0.033	0.069	0.32
Reporting fraction	f	0.0058	0.0027	0.0027
Proportionality factor between incidence of *S.* Enteritidis infection and observed incidence of invasive disease	m_X	414	-	-
Duration of cross-immunity (weeks)	$1/\chi$	442	-	-
Beginning week of increase in the duration of infectiousness or transmission rate	t_0	-	2 May 2010	8 May 2011
End week of increase in duration of infectiousness or transmission rate	t_1	-	20 July 2014	3 March 2013
Magnitude of increase in duration of infectiousness or transmission rate	m	-	1.5	2.8
BIC		6449	5966	6017

[a]$R_{0,w}$ varied with population size in Scenario 1; the values listed correspond to the range of $R_{0,w}$ between January 1996 and February 2015.

Figure S1. Seasonal variation in typhoid cases at Queen Elizabeth Central Hospital and rainfall in Blantyre, Malawi. (a) Total number of culture-confirmed *S.* Typhi cases between 1998-2014 by week of the year is plotted in dark blue, while the average weekly rainfall (in millimeters) between 2001-2010 is plotted in light blue. (b) Average weekly rainfall is plotted along with the best-fit cosine curve (with estimated mean, amplitude, and offset) in black.

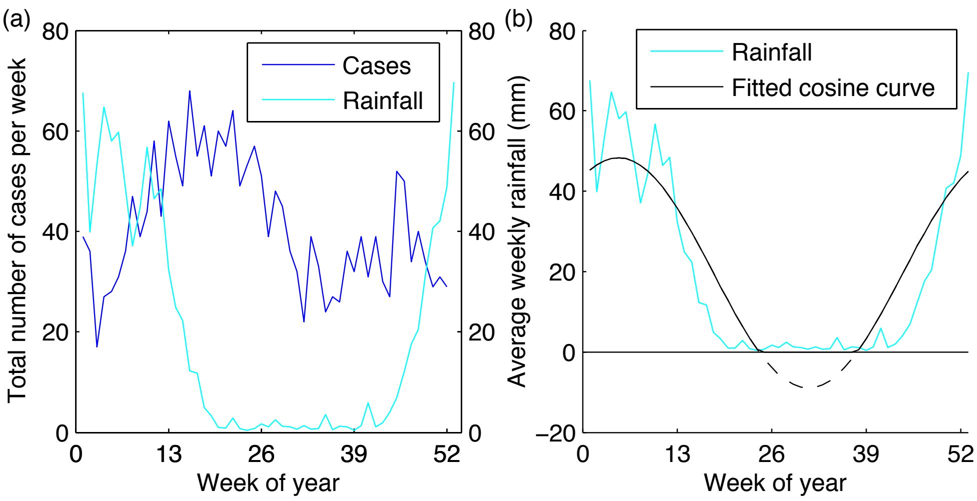

Figure S2. Population size and age distribution of Blantyre district, Malawi. (a) The model simulated population size is plotted in red, while the observed monthly population size of Blantyre district is represented by the blue x's. (b) The observed (blue) and model simulated (red) population age distribution in plotted.

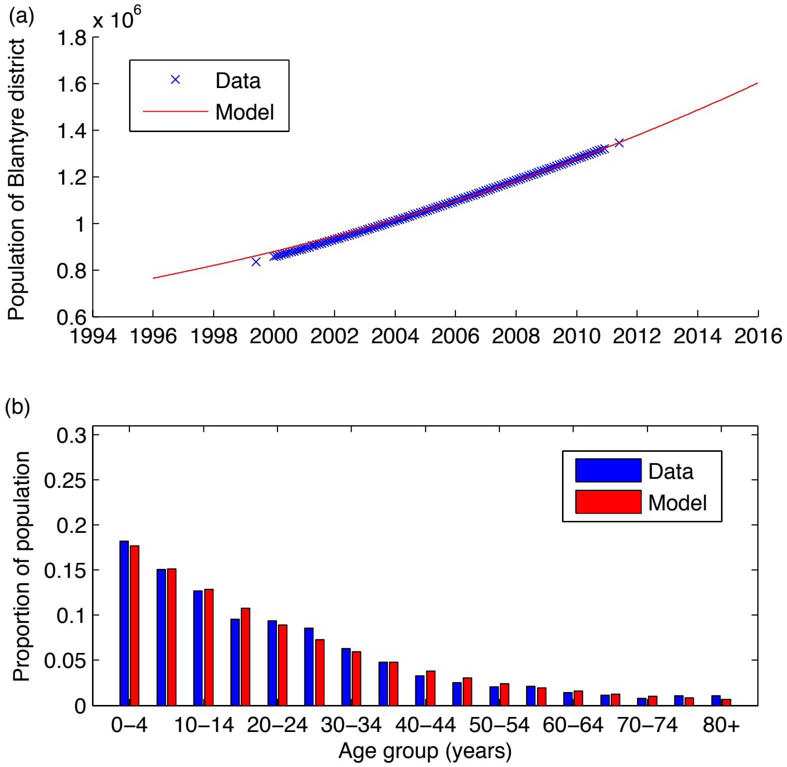

Figure S3. Contribution of acute infections and chronic carriers to the overall force of infection. The log-transformed force of infection for acute infections (black) and chronic carriers (red) are plotted for (a) Scenario 1, (b) Scenario 2, (c) Scenario 3, and (d) Scenario 4.

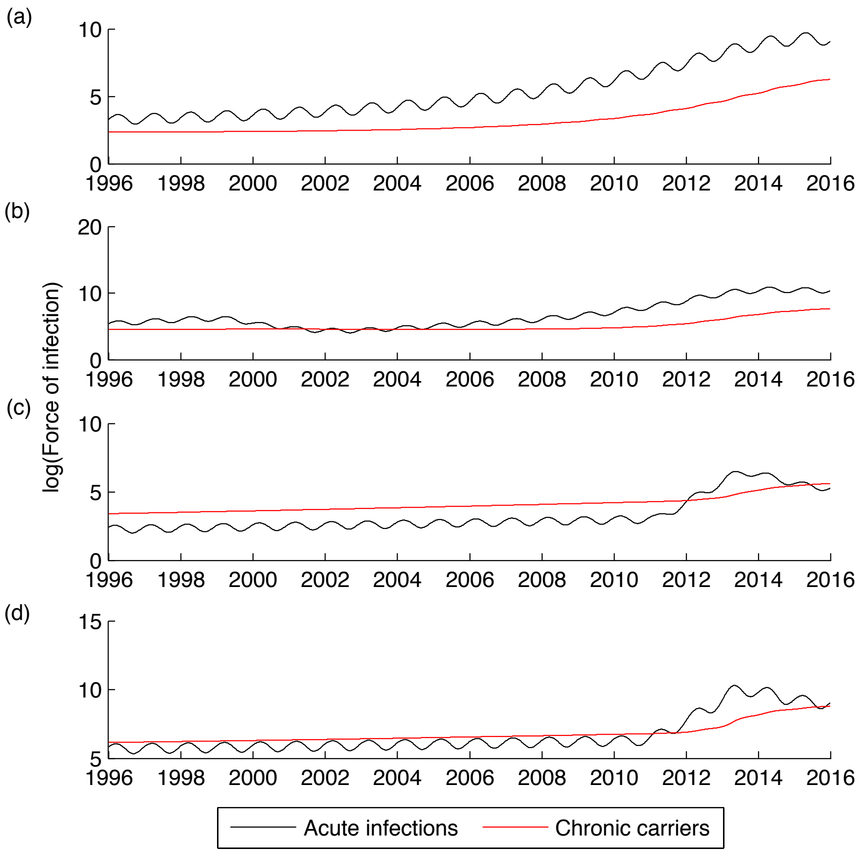

Supplement: Supplementary Data [file supp_civ710_civ710supp.pdf]
